# Investigating cardiorespiratory fitness, cerebral blood flow, and brain metabolism in epilepsy

**DOI:** 10.3389/fnetp.2026.1894335

**Published:** 2026-10-20

**Authors:** Yun Lien, Brandon S. Mitchell, Jerzy P. Szaflarski, Lawrence Ver Hoef, Thomas W. Buford, Adam M. Goodman, William R. Willoughby, Charity J. Morgan, Anna Moyana, Jennifer Pilkington, Amy R. August, Joshua T. Bennett, Jane B. Allendorfer

**Affiliations:** 1 Department of Psychology, University of Alabama at Birmingham, Birmingham, AL, United States; 2 Department of Neurology, University of Alabama at Birmingham, Birmingham, AL, United States; 3 Department of Neurobiology, University of Alabama at Birmingham, Birmingham, AL, United States; 4 Department of Neurosurgery, University of Alabama at Birmingham, Birmingham, AL, United States; 5 University of Alabama at Birmingham Epilepsy Center, Birmingham, AL, United States; 6 Birmingham Veterans Affairs Medical Center, Birmingham, AL, United States; 7 Department of Medicine, University of Alabama at Birmingham, Birmingham, AL, United States; 8 University of Alabama at Birmingham Center for Exercise Medicine, Birmingham, AL, United States; 9 Birmingham/Atlanta VA GRECC, Birmingham Veterans Affairs Medical Center, Birmingham, AL, United States; 10 Department of Radiology, University of Alabama at Birmingham, Birmingham, AL, United States; 11 Department of Biostatistics, University of Alabama at Birmingham, Birmingham, AL, United States

**Keywords:** brain metabolites, cardiorespiratory fitness, cerebral blood flow, idiopathic generalized epilepsies, network physiology, thalamocortical network

## Abstract

**Introduction:**

Idiopathic generalized epilepsies (IGEs) are characterized by seizures that propagate through widespread thalamocortical networks including the anterior cingulate cortex (ACC). Poor cardiorespiratory fitness may exacerbate neurovascular and metabolic vulnerability including heightened susceptibility to sudden unexpected death in epilepsy. We aimed to examine cardiorespiratory fitness, regional cerebral blood flow (CBF), and metabolite concentrations in patients with IGEs (PwIGEs) and to determine the effect of seizure control status on these physiological and neurochemical measures.

**Methods:**

Forty-six participants (31 PwIGEs, 15 healthy controls) underwent a graded exercise test to assess maximal oxygen uptake (VO2peak) as a measure of cardiorespiratory fitness and completed 3T MRI including multi-delayed pseudo-continuous arterial spin labeling (pCASL) to assess CBF and single-voxel spectroscopy (SVS) to assess ratios of N-acetylaspartate and glutamate/glutamine complex to total creatine (NAA/tCr and Glx/tCr, respectively) in the ACC and thalamus. Group differences and seizure control effects in PwIGEs were examined using analysis of covariance (ANCOVA).

**Results:**

VO2peak, thalamic CBF, ACC NAA/tCr, and ACC Glx/tCr did not significantly differ between groups. PwIGEs exhibited higher ACC CBF (p = 0.039) and lower thalamic NAA/tCr (p = 0.012) than healthy controls. PwIGEs with active seizures showed significantly lower thalamic Glx/tCr compared to those who were seizure-free (p = 0.003).

**Discussion:**

These findings suggest poor cardiorespiratory fitness is not epilepsy-specific and likely attributable to lifestyle and that region-specific alterations in perfusion and metabolic milieu in PwIGEs characterized by higher ACC CBF and lower thalamic NAA/tCr and Glx/tCr in those with active seizures. The multimodal neuroimaging approach provides convergent evidence of thalamocortical network dysregulation underlying IGE pathophysiology.

## 1 Introduction

Idiopathic generalized epilepsies (IGEs) comprise a group of epileptic syndromes characterized by widespread seizures that involve the entire brain without a localized seizure onset. Clinically, IGEs are categorized by a lack of macrostructural abnormalities on neuroimaging and a presumed genetic etiology (Hirsch et al., 2022). Despite accounting for approximately 20% of all epilepsies (Devinsky et al., 2024), IGEs remain markedly underrepresented in research. This gap underscores the need for integrative studies that delineate the neural and systemic physiological features of IGEs.

Unlike focal epilepsy, IGEs are linked to widespread thalamocortical network dysfunction rather than discrete seizure foci (Blumenfeld et al., 2009; Blumenfeld et al., 2003; Lindquist et al., 2023; Siman et al., 2020). The thalamus and cingulate cortex, particularly the anterior cingulate cortex (ACC), are critical regions within the thalamocortical network. The thalamus plays a critical role in the pathophysiology of IGEs, with advanced neuroimaging studies demonstrating significant gray matter reductions and neuronal loss within this region, despite the absence of lesions and visible brain damage (Blumenfeld et al., 2009; Blumenfeld et al., 2003; Bernasconi et al., 2003; Hattingen et al., 2014). Similarly, the ACC is also implicated in epileptogenic activity, as evidenced by structural abnormalities and aberrant functional networks observed in patients with IGEs (Siman et al., 2020; Braga et al., 2015; Ke et al., 2017; Paulus et al., 2015; Wei et al., 2016). Both thalamus and the ACC are involved in higher cognitive processing; the thalamus contributes to cognitive control (Wang et al., 2025), while the ACC is primarily involved in attention, error detection, decision making, and reward-guided learning (Paulus et al., 2015; Apps et al., 2016; Orr and Hester, 2012; Stevens et al., 2011). These regions comprise the thalamocortical circuit for seizure propagation and cognitive dysfunction in IGEs, providing a mechanistic rationale for region-specific imaging analyses.

Advanced neuroimaging modalities, such as arterial spin labeling (ASL) and magnetic resonance spectroscopy (MRS), allow for the noninvasive quantification of cerebral perfusion and neurochemical alterations, respectively, and provide unique insight into the brain network physiology of healthy and neurological populations. ASL quantifies cerebral blood flow (CBF) *in vivo* (Alsop et al., 2015; Grade et al., 2015), and previous studies have reported decreased thalamic CBF but increased frontal CBF in patients with IGEs compared with healthy controls (Chen G. et al., 2022; Sone et al., 2017). Altered CBF has also been linked to cognitive impairments in IGEs (Blumenfeld et al., 2009; Blumenfeld et al., 2003; Lien et al., 2025), suggesting that regional perfusion may serve as a potential biomarker of disease severity. MRS enables *in vivo* quantification of brain metabolite concentrations (Barker et al., 2001). N-acetylaspartate (NAA) is a marker of neuronal integrity (Moffett et al., 2007), whereas the glutamate-glutamine complex (Glx) reflects excitatory neurotransmission and cognitive processing (Petroff, 2002). Previous studies have shown reduced thalamic NAA levels in IGEs (Bernasconi et al., 2003; Cevik et al., 2016; Doelken et al., 2010; Fojtikova et al., 2006; Helms et al., 2006; Lin et al., 2009; Savic et al., 2004), but evidence for Glx alterations in the thalamus or metabolite changes in the ACC remains limited. Despite emerging evidence of perfusion and metabolic abnormalities, no prior study has jointly examined these multimodal features in IGEs.

Beyond neural alterations, systemic physiological factors may also differ in IGEs. Cardiorespiratory fitness, commonly indexed by peak oxygen uptake (VO_2_peak), reflects the integrated capacity of the cardiovascular and respiratory systems to deliver oxygen during exercise (Garber et al., 2011). As a brain-body axis, this capacity directly influences cerebral oxygen and nutrients delivery; consequently, higher VO_2_peak is associated with greater CBF in some brain regions, particularly within thalamus (Delp et al., 2001). Patients with epilepsy often exhibit reduced physical activity and lower VO_2_peak compared with healthy individuals (Vancini et al., 2010; Vancini et al., 2015; Volpato et al., 2017), potentially contributing to cardiovascular risk, cognitive deficits, and heightened susceptibility to sudden unexpected death in epilepsy (SUDEP) (Allendorfer and Arida, 2018; Johnson et al., 2020). However, some studies have found comparable VO_2_peak levels between healthy participants and patients with epilepsy (de Lima et al., 2011; van den Bongard et al., 2022). Clarifying whether IGEs specifically involve diminished fitness capacity–beyond low levels of self-reported physical activity (Popp et al., 2021; Sharma et al., 2024) – is essential, as impaired VO_2_peak may exacerbate neurovascular and metabolic vulnerability.

While perfusion, metabolic, and cardiorespiratory alterations are recognized features of epilepsy, these alterations have only been studied in isolation. Furthermore, evidence focusing on these features in IGEs remains scarce. To address these gaps, we applied a multimodal framework integrating ASL, MRS, and VO_2_peak assessment to characterize the physiological and neural features of IGEs. We compared VO_2_peak, regional CBF, and metabolite concentrations (NAA and Glx) between patients with IGEs and healthy controls. Further, we examined whether seizure-control status modulated these measures. We hypothesized patients with IGEs would exhibit lower VO_2_peak, decreased thalamic and increased ACC CBF, and reduced thalamic NAA with elevated Glx levels relative to controls. This integrative approach provides a comprehensive physiological and neuroimaging characterization of IGEs, offering insights that may inform future biomarkers and therapeutic monitoring in clinical practice.

## 2 Methods

### 2.1 Participants

Adult patients with IGEs were recruited from the University of Alabama at Birmingham (UAB) Epilepsy Center and Neurology clinics as part of a blinded randomized controlled trial (RCT) of combined endurance and resistance exercise training (NCT04959019; baseline data only). Healthy controls (HCs) were recruited from the UAB community as part of an endurance training pilot study (baseline data only). Inclusion criteria for patients with IGEs included: (1) 18–65 years old; (2) clinician-confirmed diagnosis of IGEs; (3) no contraindications to MRI; (4) no major comorbid medical conditions; and (5) physical activity levels below American Heart Association (AHA) recommendations of 150 min/week of moderate intensity endurance exercise. Exclusion criteria were degenerative or metabolic disorders, abnormal physical examination by a study clinician (JPS or LVH), pregnancy, intellectual disability, uncontrolled hypertension, cardiovascular disease, and concurrent enrollment in another intervention trial. HCs met the same eligibility requirements except for the epilepsy diagnosis. Study protocols were approved by the UAB Institutional Review Board (IRB); procedures included a physical exam, maximal graded exercise test (GXT) and an MRI scan. Written informed consent was obtained from all participants prior to participation. The Baecke Questionnaire of Habitual Physical Activity (BQ) assessed self-reported physical activity in leisure time. To mitigate potential bias arising from differential sources, we employed a standardized assessment protocol. All participants underwent standardized procedures: (1) same inclusion/exclusion criteria except for required IGE diagnosis in the IGE group, (2) the study physicians (JPS and LVH) conducting physical exams for both studies, (3) VO_2_peak testing for both studies conducted by UAB Center for Exercise Medicine (UCEM) staff using their standardized exercise test protocols, and (4) 3T MRI acquisition with the same scanner, sequences, acquisition parameters, and voxel placement procedures. Moreover, to examine the impact of seizure control, participants with IGE were stratified into two subgroups based on their self-reported seizure history in the 6 months prior to enrollment. The 6-month time frame for seizure-freedom was utilized per the recommendations of the study’s epilepsy specialists (JPS and LVH), as this criterion reflects seizure management and control that is stable enough to allow a patient to legally drive, and driving positively impacts patient independence and quality of life (Drazkowski, 2007). The 6-month time frame for seizure freedom is also consistent with prior studies in patients with IGEs (Popp et al., 2021; Bolden et al., 2018). Participants who reported no seizures of any type during this interval were classified as seizure-free (IGE–), whereas those who experienced one or more seizures were classified as having active seizures (IGE+).

### 2.2 Graded exercise test (GXT)

Participants underwent a maximal graded exercise test (GXT) at the UCEM facility. Following a standardized ramp protocol on a Via Spring 150 P-calibrated cycle ergometer, the test started at a work rate of 25 W and increased in 25 W increments every 2 min at a cadence of 50 rpm. To evaluate maximal effort, respiratory exchange ratio, blood pressure, and peak heart rate were continuously monitored. Participants also reported ratings of perceived exertion (RPE) on a 0.5–10 scale (Borg, 1982), where maximal exertion (RPE = 10) provided subjective confirmation of maximal effort. Oxygen uptake (VO_2_) and carbon dioxide production were measured breath-by-breath using a CareFusion indirect calorimetry system and averaged over 30-s intervals. Peak oxygen uptake (VO_2_peak), defined as the highest VO_2_ achieved during the test, was calculated from the final two measurements and served as the primary index of cardiorespiratory fitness for the following analysis. Two participants were excluded from VO_2_peak analysis due to failing to complete the test.

### 2.3 Magnetic resonance imaging (MRI)

Participants underwent an MRI scan on a 3T Siemens MAGNETOM Prisma scanner with a 20-channel head coil at the UAB Civitan International Neuroimaging Laboratory/Research MRI Core.

High-resolution T1-weighted images were acquired in the sagittal plane using an MPRAGE sequence (repetition time [TR] = 2400 ms; echo time [TE] = 2.22 ms; inversion time [TI] = 1000 ms; flip angle [FA] = 8°; voxel size = 0.8 mm isotopic).

Arterial spin labeling (ASL) data were acquired using a five-delay pseudo-continuous arterial spin labeling (pCASL) sequence with background-suppressed 3D turbo gradient and spin echo (TGSE) readouts (TR = 4100 ms; TE = 31.7 ms; FA = 120°; bolus duration = 700 ms; labeling duration = 1800 ms; post labeling delay (PLD) = 500/1000/1500/2000/2500 ms; tag-control pairs per delay = 2/2/2/3/3; voxel size = 3.5 mm isotropic). An M_0_ image was acquired with the same readout using a PLD of 2000 ms for calibration. Both cerebral blood flow (CBF) and arterial transit time (ATT) were estimated by a multi-delay kinetic model.

Single-voxel spectroscopy (SVS) data were collected with a point-resolved spectroscopy (PRESS) sequence (TR = 1500 ms; TE = 80 ms, FA = 90°). A single 20 × 40 × 10 mm^3^ voxel was localized on the bilateral dorsal anterior cingulate cortex (ACC) extending into part of the middle cingulate cortex (Figure 1A) (Orr and Hester, 2012; Chen YC. et al., 2022; Modirrousta and Fellows, 2008; Tolomeo et al., 2016) and a 40 × 20 × 20 mm^3^ voxel was localized on the bilateral thalamus (Figure 1B) for each individual. Unsuppressed water reference scans with the same voxel placement were used for eddy-current correction and metabolite quantification.

**FIGURE 1:**
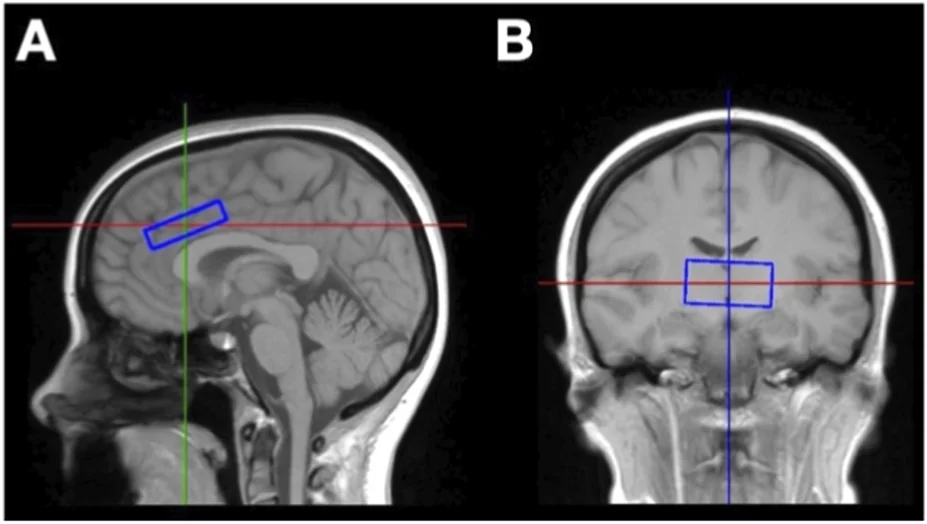
Representative voxel placement for single-voxel spectroscopy in the anterior cingulate cortex $20\times 40\times 10\;{mm}^{3}$ ; **(A)** and thalamus $40\times 20\times 20\;{mm}^{3}$ ; **(B)**.

### 2.4 Data analysis

#### 2.4.1 pCASL data processing

pCASL images were processed with FSL 6.0.3 (Functional MRI of the Brain Software Library) (Jenkinson et al., 2012) and its built-in toolbox, BASIL (Chappell et al., 2011; Chappell et al., 2009; Chappell et al., 2010; Groves et al., 2009). Perfusion images were generated via tag-control subtraction. Motion correction was performed for both perfusion and M_0_ images using MCFLIRT. The perfusion images were co-registered to T1-weighted images and underwent partial volume correction to generate separate perfusion maps for gray matter and white matter (Chappell et al., 2011). For CBF quantification, the M_0_ image calibrated the perfusion images to produce CBF maps in absolute units (ml/100 g/min). Participants were excluded for ghosting artifacts (n = 1) or high perfusion variance (variance >400; n = 7) (Chappell et al., 2023; Fallatah et al., 2018). Based on these criteria, eight participants were excluded from CBF analyses. Region analyses used the Harvard-Oxford standard atlas to extract the mean CBF values weighted by voxel-wise precision (1/standard deviation) estimates in the ACC and thalamus.

#### 2.4.2 SVS data processing

SVS data were analyzed by Osprey toolbox (Oeltzschner et al., 2020) with the LCModel fitting algorithm (Provencher, 1993). Post-processing included eddy-current correction, frequency-and-phase alignment, water removal, frequency referencing, and model fitting. Metabolite and water spectra were modeled using linear-combination with basis sets to estimate individual metabolite concentration. Processed spectra were co-registered to T1-weighted images for voxel localization, and gray matter, white matter, and cerebrospinal fluid fractions were extracted for tissue correction. Glx and NAA concentration were normalized to total creatine (tCr), which is widely used as a reliable reference due to relatively stable levels in epilepsy (Savic et al., 2004). Data were excluded if the full width at half maximum (FWHM) of creatine exceeded 10 Hz or signal-noise ratio of creatine is smaller than 15 (Marjańska and Terpstra, 2021; van der Graaf et al., 2008). Seven participants were excluded from SVS analysis based on these criteria.

### 2.5 Statistical analysis

Statistical analysis was performed using SPSS (IBM SPSS Statistics, version 30). Demographic comparisons between groups were conducted by one-way analysis of variance (ANOVA) for age and chi-square tests for sex distributions. To determine appropriate covariates, Pearson correlation analyses and independent t-tests assessed the influence of age and sex on VO_2_peak, regional CBF, and brain metabolites (NAA/tCr and Glx/tCr). Analyses of covariate (ANCOVAs), adjusting for covariates, examined differences between HCs and patients with IGEs, as well as the effect of seizure control (IGE–vs. IGE+). Post-hoc comparisons were performed using Tukey’s honestly significant difference (HSD). Effect sizes are reported as partial eta-squared ( $\eta_{p}^{2}$ ). All p-values are two-sided and p < 0.05 was considered statistically significant.

## 3 Results

Forty-six participants were included in the study, comprising thirty-one patients with IGEs and fifteen HCs (Table 1). The BQ physical activity in leisure time did not differ significantly between healthy and IGEs (HC: EMM = 2.31, 95%CI [1.75, 2.87]; IGE: EMM = 2.18, 95%CI [1.79, 2.56]; F (1,42) = 0.15, p = 0.696; controlling for age and sex), supporting comparable baseline activity. Most patients with IGEs (30 of 31) were currently being treated with one or more antiseizure medications (ASMs); one patient who was seizure-free was not taking any ASM at the time of the study. Sample sizes for each analysis varied based on the data quality criteria in the methods.

**TABLE 1:** Demographic data for healthy controls (HC) and patients with idiopathic generalized epilepsies (IGE). Patients with IGE were further divided into two groups–seizure-free IGE (IGE–), and IGE with active seizures (IGE+).

​	​	IGE: ALL		
​	HC	All	IGE-	IGE+
N	15	31	17	14
Age, years	37.3 (11.0)	29.7 (9.9)	29.8 (9.9)	29.5 (10.2)
Sex, female, N (%)	8 (53%)	18 (58%)	8 (47%)	10 (71%)
Duration of epilepsy, years	-	14.1 (10.7)	14.9 (9.9)	13.2 (11.9)
Number of seizures in past 6 months	-	0.8 (1.1)	0.0 (0.0)	1.9 (0.9)
Number of antiseizure medications	-	1.7 (0.9)	1.6 (0.9)	1.9 (0.8)
Monotherapy/Polytherapy, N (%)	-	12/18 (39%/58%)	7	5
Types of antiseizure medications				
Sodium valproate	-	5 (16%)	3	2
Clonazepam	-	3 (10%)	1	2
Levetiracetam	-	15 (48%)	10	5
SSRI	-	1 (3%)	1	0
Aripiprazole	-	1 (3%)	1	0
Lamotrigine	-	13 (42%)	6	7
Cannabidiol	-	1 (3%)	1	0
Oxcarbazepine	-	4 (13%)	0	4
Brivaracetam	-	1 (3%)	0	1
Benzodiazepine	-	1 (3%)	1	0
Topiramate	-	2 (6%)	1	1
Zonisamide	-	2 (6%)	1	1
Phenobarbital	-	1 (3%)	0	1
Lacosamide	-	2 (3%)	2	0
Pregabalin	-	1 (3%)	0	1
Amisulpride	​	1 (3%)	0	1

N, number of participants. Data are presented as mean (standard deviation) for continuous variables (Age, Duration of epilepsy, Number of seizures in past 6 months, and Number of antiseizure medications) or number (percentage) for categorical variables (Sex, Monotherapy/Polytherapy, and Types of antiseizure medications). Monotherapy/Polytherapy refers to treatment with single vs. multiple antiseizure medications at the time of the study. Note that one of the seizure-free patients was not taking any antiseizure medications. Types of antiseizure medications lists the number of patients taking each type of medication. Percentages for types of antiseizure medications do not sum to 100% due to patients with polytherapy.

### 3.1 Group comparisons in VO_2_peak

Forty-four participants completed cardiorespiratory fitness testing (HCs: n = 14; IGE–: n = 16; IGE+: n = 14). There were no significant differences in the age or sex between groups (all ps > 0.05). Age did not correlate with VO_2_peak (r = −0.14, p = 0.360), whereas a significant sex difference was observed (t (42) = −2.91, p = 0.006; Supplementary Table S1). Though age was not correlated to the VO2_2_peak, previous research demonstrated that cardiorespiratory fitness was influenced by age (Letnes et al., 2023; Talbot et al., 2000). Thus, both age and sex were included as covariates in subsequent analyses.

After controlling for age and sex, ANCOVA revealed no significant group differences in VO_2_peak (F (1, 40) = 1.68, p = 0.203, $\eta_{p}^{2}$ = 0.04). Estimated marginal means (EMMs) for VO_2_peak were comparable between the IGEs (20.41 mL·kg^-1^·min^-1^, 95% CI [18.32, 22.49]) and HCs (22.85 mL·kg^-1^·min^-1^, 95% CI [19.75, 25.95]; Figure 2A).

**FIGURE 2:**
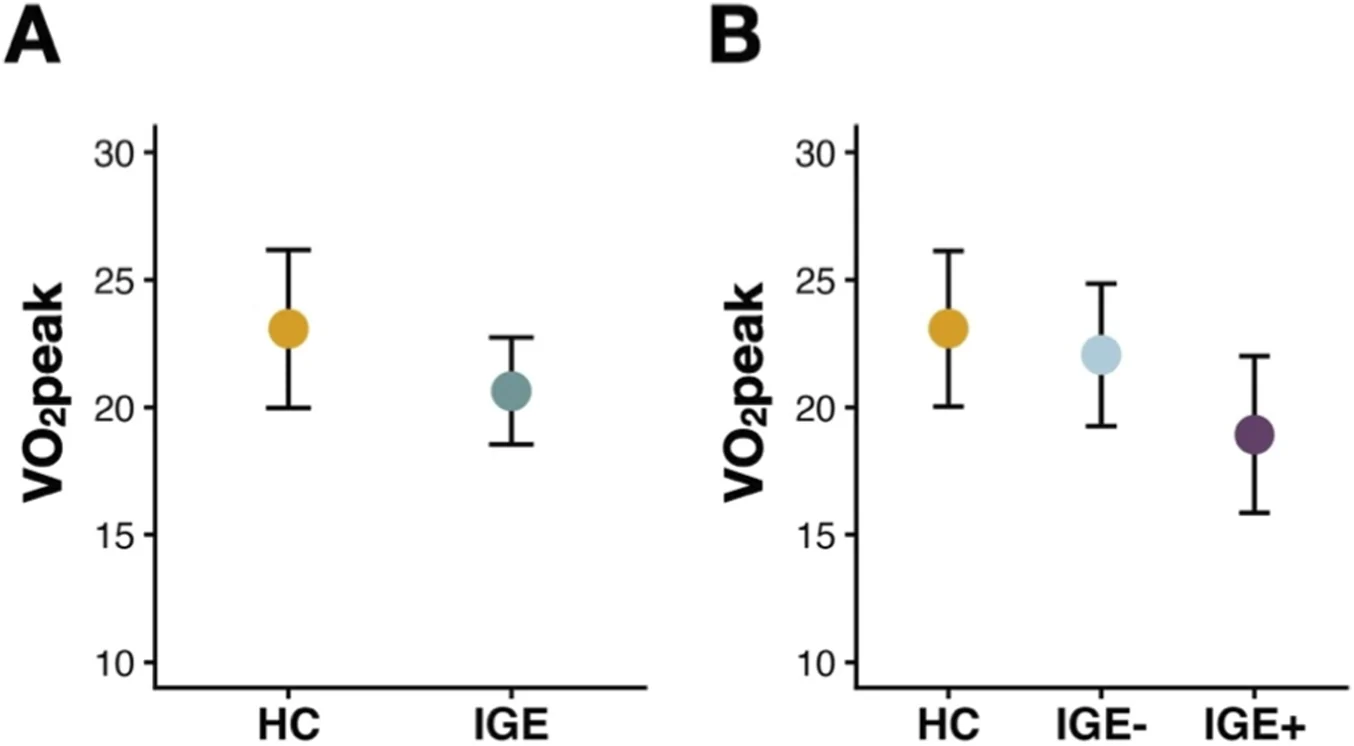
Estimated marginal means (EMMs) of peak oxygen uptake (VO_2_peak; mL/kg/min) during the GXT tests across groups. Points represent the adjusted group means with 95% confidence intervals. **(A)** Comparison between healthy controls (HC) and patients with idiopathic generalized epilepsies (IGE). **(B)** Comparisons among HC, seizure-free IGE (IGE–), and active-seizure IGE (IGE+).

To examine the effect of seizure control, ANCOVA compared HC, IGE–, and IGE + subgroups. The main effect of seizure control on VO_2_peak was not significant (F (2, 39) = 2.02, p = 0.146, $\eta_{p}^{2}$ = 0.09; HCs: EMM = 22.88 mL·kg^-1^·min^-1^, 95% CI [19.83, 25.94]; IGE–: EMM = 21.85 mL·kg^-1^·min^-1^, 95% CI [19.04, 24.67]; IGE+: EMM = 18.72 mL·kg^-1^·min^-1^, 95% CI [15.68, 21.76]; Figure 2B).

### 3.2 Group comparisons in CBF

Thirty-eight participants (HCs: n = 12; IGE–: n = 15; IGE+: n = 11) were included in the CBF comparisons. No significant group differences were found for age or sex distribution (all p > 0.05). ACC CBF was significantly negatively correlated with age (r = −0.61, p < 0.001), while thalamic CBF showed no association (r = −0.15, p = 0.383). ACC CBF did not differ by sex (t (36) = 0.71, p = 0.482; Supplementary Table S1), but thalamic CBF was significantly higher in females than in males (t (36) = 2.21, p = 0.034; Supplementary Table S1). Age and sex were included as covariates for ANCOVA analyses.

For ACC CBF, ANCOVA revealed a significant group effect (F (1, 34) = 4.61, p = 0.039, $\eta_{p}^{2}$ = 0.12; Figure 3A). EMMs showed significant higher ACC CBF in the IGEs (59.61 mL · 100 g^-1^ min^-1^, 95% CI [56.02, 63.19]) than in HCs (52.72 mL · 100 g^-1^ min^-1^, 95% CI [47.40, 58.04]; Δ = 6.89, p = 0.039). These findings indicate elevated ACC CBF in IGEs relative to HCs, independent of age and sex.

**FIGURE 3:**
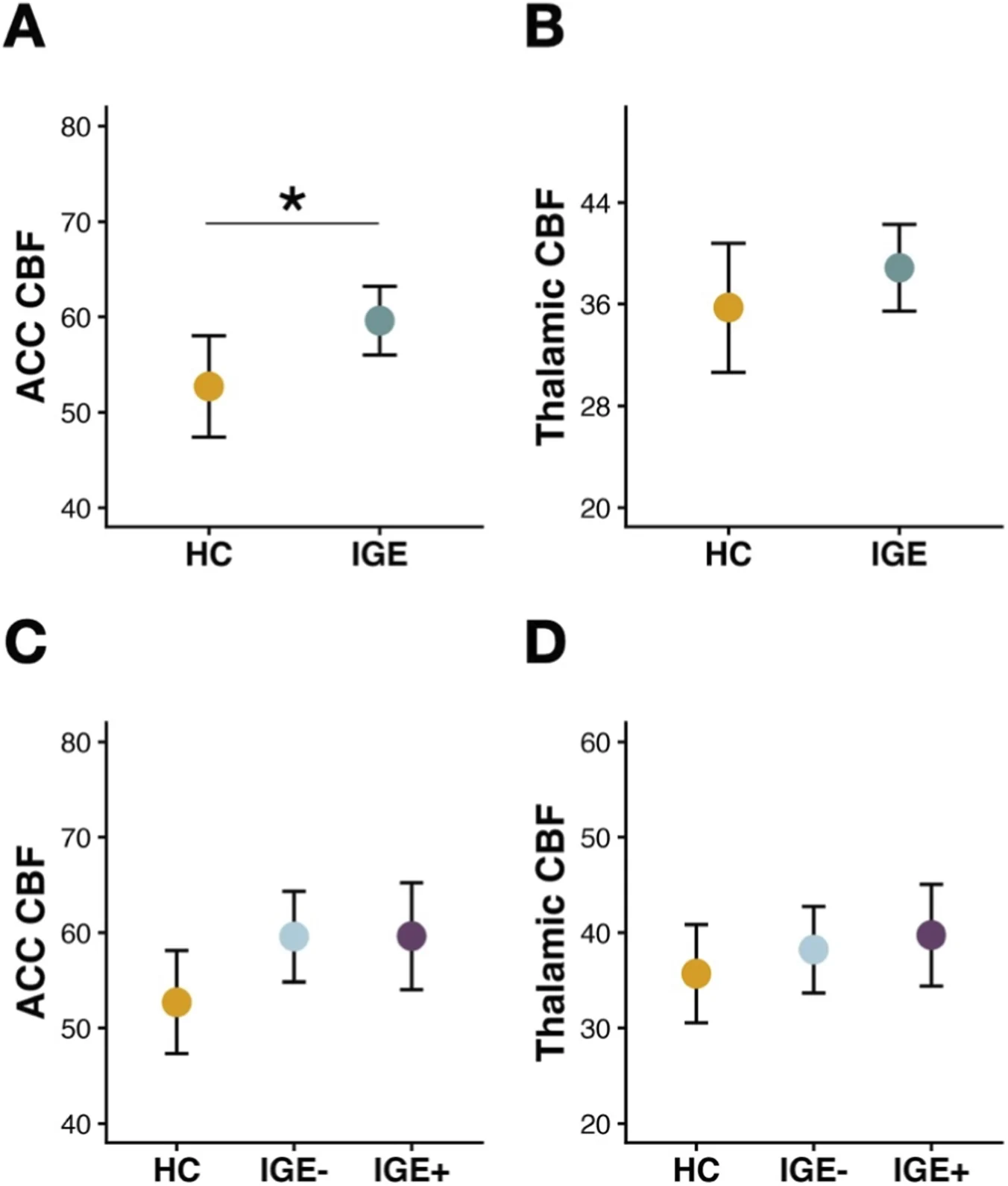
Estimated marginal means (EMMs) of cerebral blood flow (CBF; ml/100 g/min) with 95% confidence intervals in the anterior cingulate cortex (ACC) and thalamus. **(A,B)** Group comparisons between healthy controls (HC) and patients with idiopathic generalized epilepsies (IGE) in the ACC and thalamus, respectively. **(C,D)** Group comparisons among HC, seizure-free IGE (IGE–), and active-seizure IGE (IGE+) in the ACC and thalamus, respectively. *p < 0.05.

For thalamus CBF, no significant group effect was observed (F (1, 34) = 1.05, p = 0.313, $\eta_{p}^{2}$ = 0.03; Figure 3B; HCs: EMM = 35.72 mL · 100 g^-1^ min^-1^, 95% CI [30.64, 40.80]; IGEs: EMM = 38.86 mL · 100 g^-1^ min^-1^, 95% CI [35.44, 42.28]). Thus, group differences in CBF were confined to the ACC.

ANCOVAs revealed no significant main effect of seizure control on ACC CBF (F (2, 33) = 2.24, p = 0.123, $\eta_{p}^{2}$ = 0.12; HCs: EMM = 52.72 mL · 100 g^-1^ min^-1^, 95% CI [47.32, 58.12]; IGE–: EMM = 59.59 mL · 100 g^-1^ min^-1^, 95% CI [54.84, 64.33]; IGE+: EMM = 59.64 mL · 100 g^-1^ min^-1^, 95% CI [54.05, 65.23]; Figure 3C) or thalamic CBF (F (2, 33) = 0.61, p = 0.548, $\eta_{p}^{2}$ = 0.04; HCs: EMM = 35.71 mL · 100 g^-1^ min^-1^, 95% CI [30.57, 40.85]; IGE–: EMM = 38.22 mL · 100 g^-1^ min^-1^, 95% CI [33.71, 42.74]; IGE+: EMM = 39.75 mL · 100 g^-1^ min^-1^, 95% CI [34.42, 45.07]; Figure 3D).

Overall, CBF in the ACC was inversely related to age and was higher in IGEs than HCs, and thalamic CBF was significantly higher in females than in males, whereas CBF was not significantly affected by seizure control in either region.

### 3.3 Group comparisons in brain metabolites levels

Thirty-nine participants (HCs: n = 14; IGE–: n = 14; IGE+: n = 11) were included in the metabolite analysis. No significant group differences existed for age or sex (all ps > 0.05). Metabolite ratios did not correlate with age (all ps > 0.05). A significant sex difference was observed only for ACC Glx/tCr (t (37) = −2.19, p = 0.035; Supplementary Table S1), and sex was included as a covariate in following analysis.

In the ACC, no significant main effect of group was found for NAA/tCr (F (1, 36) = 1.62, p = 0.211, $\eta_{p}^{2}$ = 0.04; HCs: EMM = 1.23, 95% CI [1.17, 1.29]; IGE: EMM = 1.18, 95% CI [1.13, 1.23]; Figure 4A) or Glx/tCr (F (1, 36) = 0.25, p = 0.619, $\eta_{p}^{2}$ = 0.01; HCs: EMM = 1.09, 95% CI [0.99, 1.18]; IGE: EMM = 1.05, 95% CI [0.98, 1.13]; Figure 4B).

**FIGURE 4:**
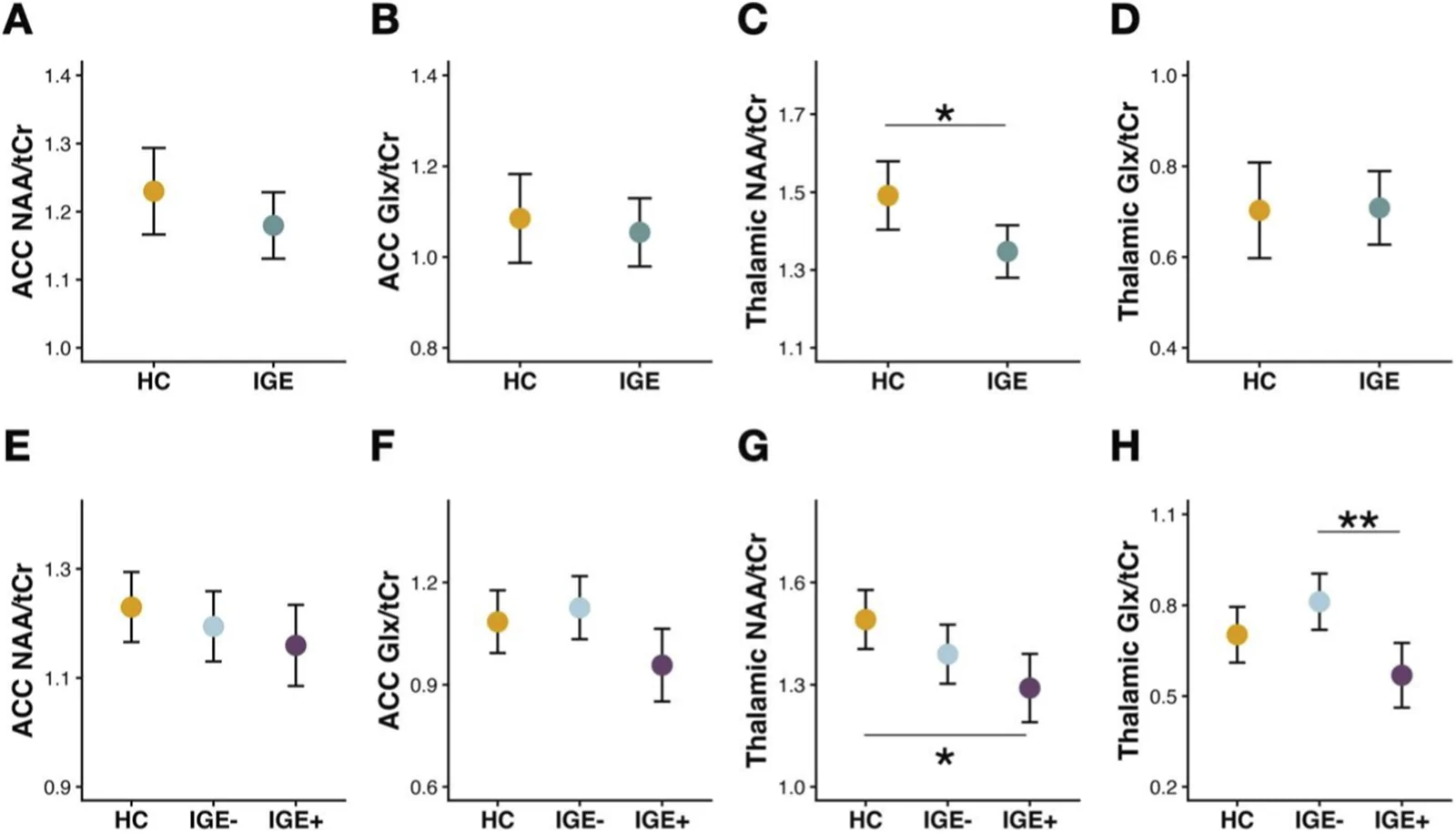
Estimated marginal means (EMMs) of brain metabolites with 95% confidence intervals in the anterior cingulate cortex (ACC) and thalamus. **(A,B)** Group comparisons of N-acetylaspartate to total creatine ratio (NAA/tCr) and glutamate-glutamine complex to total creatine ratio (Glx/tCr) between healthy controls (HC) and patients with idiopathic generalized epilepsies (IGE) in the ACC. **(C,D)** Corresponding comparisons between HC and IGE in the thalamus. **(E,F)** Group comparisons of NAA/tCr and Glx/tCr among HC, seizure-free IGE (IGE–), and active-seizure IGE (IGE+) in the ACC. **(G,H)** Corresponding group comparisons among HC, IGE–, and IGE+ in the thalamus. *: p < 0.05; **: p < 0.01.

In the thalamus, a significant group effect was detected for NAA/tCr (F (1, 36) = 6.94, p = 0.012, $\eta_{p}^{2}$ = 0.16, Figure 4C), with significant lower ratios in IGEs (EMM = 1.35, 95% CI [1.28, 1.41]) than in HCs (EMM = 1.49, 95% CI [1.40, 1.58]; Δ = −0.14, p = 0.012). In contrast, no group difference was found for Glx/tCr (F (1, 37) = 0.01, p = 0.934, $\eta_{p}^{2}$ = 0.00; Figure 4D; HCs: EMM = 0.70, 95% CI [0.60, 0.81]; IGEs: EMM = 0.71, 95% CI [0.63, 0.79]).

To examine the effect of seizure control, ANCOVA showed no significant main effect of seizure control on ACC NAA/tCr (F (2, 35) = 1.06, p = 0.357, $\eta_{p}^{2}$ = 0.06; HCs: EMM = 1.23, 95% CI [1.17, 1.29]; IGE–: EMM = 1.19, 95% CI [1.13, 1.26]; IGE+: EMM = 1.16, 95% CI [1.09, 1.23]; Figure 4E) or ACC Glx/tCr (F (2, 35) = 3.15, p = 0.055, $\eta_{p}^{2}$ = 0.15; HC: EMM = 1.09, 95% CI [0.99, 1.18]; IGE–: EMM = 1.13, 95% CI [1.03, 1.22]; IGE+: EMM = 0.96, 95% CI [0.85, 1.06]; Figure 4F).

In thalamus, the effect of seizure control on NAA/tCr ratios was significant (F (2, 35) = 4.78, p = 0.015, $\eta_{p}^{2}$ = 0.21; HCs: EMM = 1.49, 95% CI [1.41, 1.58]; IGE–: EMM = 1.39, 95% CI [1.30, 1.48]; IGE+: EMM = 1.29, 95% CI [1.19, 1.39]; Figure 4G). Post hoc pairwise tests indicated that the IGE + had lower thalamic NAA/tCr than HCs (Δ = −0.20, t = −2.9, p = 0.017), whereas other pairwise contrasts were nonsignificant. For Glx/tCr, the ANCOVA showed a significant main effect of seizure control (F (2, 35) = 6.22, p = 0.005, $\eta_{p}^{2}$ = 0.26; HCs: EMM = 0.70, 95% CI [0.61, 0.79]; IGE–: EMM = 0.81, 95% CI [0.72, 0.90]; IGE+: EMM = 0.57, 95% CI [0.46, 0.68]; Figure 4H). Post hoc analyses showed that the IGE + had lower Glx/tCr than IGE– (Δ = −0.24, t = −3.47, p = 0.004), with no other significant contrasts.

## 4 Discussion

This study demonstrated that patients with IGEs exhibit higher ACC CBF and lower thalamic NAA/tCr compared with healthy controls. Notably, the metabolite levels in the thalamus varied by seizure control status. Patients with active seizures demonstrated significantly lower thalamic NAA/tCr than healthy controls and significantly lower thalamic Glx/tCr than seizure-free patients. No significant difference in VO_2_peak was observed between groups.

In our study, no significant group difference in VO_2_peak was found between patients with IGEs and healthy controls, although there was a trend toward lower VO_2_peak in IGEs, suggesting relatively worse cardiorespiratory fitness. Moreover, VO_2_peak did not differ by seizure control. One possible explanation for these null findings is that participants in both IGEs and HCs groups did not meet AHA physical activity recommendations. People with epilepsy generally engage in less physical activity due to seizure-related risks (Johnson et al., 2020). To minimize this bias, we recruited healthy controls with below-recommended activity levels, which likely reduced intergroup variability in VO_2_peak. Similar findings have been reported when physical-activity levels were matched (Volpato et al., 2017; de Lima et al., 2011). The comparable VO_2_peak across groups, each recruited with similarly low physical activity, raises the possibility that previous studies showing group differences in cardiorespiratory fitness may be attributed to lifestyle-related physical inactivity rather than epilepsy, although we cannot entirely exclude any epilepsy-specific impacts. This interpretation remains tentative due to the cross-sectional design, the absence of objective physical activity measurement, and the potential influence of antiseizure medications on exercise capacity. Given that impaired VO_2_peak may exacerbate neurovascular and metabolic vulnerability in patients with epilepsy including heightened susceptibility to SUDEP (Allendorfer and Arida, 2018; Johnson et al., 2020), the current study results suggesting that lifestyle physical activity modification may minimize such vulnerabilities provides a promising avenue of risk reduction. Future research should also explicitly quantify leisure-time activity (e.g., using activity trackers) to improve accuracy when evaluating physical activity levels in epilepsy populations.

We found increased ACC CBF but no significant thalamic CBF difference in patients with IGEs. ACC hyperperfusion is consistent with prior observations of frontal overactivation, which is positive correlated to the cognitive-related brain networks (Centeno et al., 2012; Xu et al., 2021). However, the lack of thalamic hypoperfusion in IGEs compared with HCs is inconsistent with earlier reports showing reduced thalamic CBF in patients with IGEs who had uncontrolled seizures or were drug-naïve (Sone et al., 2017; Tae et al., 2007). Notably, previous studies used voxel-wise comparisons and showed increases in CBF in only part of the thalamus. The current study utilized anatomically defined region of interest analysis to extract the mean CBF in the thalamus, which might not reflect alterations within specific regions of the thalamus (Kay et al., 2013; Tyvaert et al., 2009). It is also possible that our groups being similar in cardiorespiratory fitness may have contributed to the lack of thalamic CBF differences, since increased VO_2_peak has been associated with increased CBF, particularly in areas involved in locomotion control such as the thalamus (Delp et al., 2001). Factors such as VO_2_peak that may affect CBF in IGEs should be further investigated in future studies.

We also observed that age modulated regional CBF, with divergent effects across brain regions. These findings align with evidence that global CBF progressively declines with age (Claassen et al., 2021), particularly in frontal and temporal regions (Chen et al., 2011; Jefferson et al., 2017; Lu et al., 2011), due to cerebral atrophy and high arterial blood pressure. The wide age distribution in our cohort may have increased variability, as older participants are more likely to exhibit vascular comorbidities that impair CBF autoregulation (Claassen et al., 2021). Furthermore, antiseizure medications such as lamotrigine have been shown to alter cerebral perfusion (Joo et al., 2006). Although medication histories were recorded, we did not examine medication effects due to the heterogeneity of drug regimens. Future work should address medication influences and vascular risk factors to clarify the mechanisms underlying altered CBF in IGEs.

In the present study, thalamic NAA/tCr was lower in patients with IGEs than in healthy individuals, and NAA/tCr differed based on seizure control. This reduction may reflect diminished neuronal integrity in the thalamus. Our findings are consistent with prior evidence implicating thalamic neuronal dysfunction in IGEs (Bernasconi et al., 2003; Hattingen et al., 2014; Cevik et al., 2016; Doelken et al., 2010; Fojtikova et al., 2006; Helms et al., 2006; Lin et al., 2009; Savic et al., 2004; Abedi-Firouzjah et al., 2020). However, few studies have examined the influence of seizure control on thalamic NAA levels. Prior evidence indicates that thalamic NAA decreases with duration of epilepsy (Bernasconi et al., 2003). Our finding of reduced thalamic NAA/tCr in patients with poorly-controlled epilepsy relative to controls suggests that persistent seizures may be associated with thalamic neuronal dysfunction.

We further found a significant main effect of seizure control on thalamic Glx/tCr ratios, in which patients with poorly-controlled epilepsy showed significantly lower Glx/tCr than those who were seizure-free. In contrast, no significant group difference was observed in ACC Glx/tCr. Prior literature is mixed, with some studies reporting elevated Glx/tCr in frontal and subcortical regions (Doelken et al., 2010; Helms et al., 2006; Chowdhury et al., 2015; de Araujo Filho et al., 2009; Simister et al., 2003; Zhang et al., 2016) and others showing inconsistent findings (Hattingen et al., 2014; Cevik et al., 2016; Lin et al., 2009). Our findings suggest that thalamic Glx levels may be sensitive to seizure control status. Such alterations may reflect disrupted glutamate-glutamine cycling and excitatory-inhibitory imbalance. In the healthy brain, glutamate released during synaptic transmission is rapidly taken up by astrocytes and converted to glutamine, which is then recycled to neurons. GABA, the major inhibitory transmitter in the central nervous system (Petroff, 2002), is often deficient in IGEs (Rowley et al., 2012; Snodgrass, 1992; Zhang et al., 2021). In epilepsy, this metabolic loop that maintains excitatory-inhibitory balance becomes dysregulated, leading to sustained hyperexcitability (Barker-Haliski and White, 2015; Coulter and Eid, 2012). Furthermore, excessive glutamatergic activity and impaired GABAergic inhibition contribute to the excitatory-inhibitory imbalance characteristic of IGE (Chowdhury et al., 2015; Zhang et al., 2021; Fritschy, 2008; Sears and Hewett, 2021). However, the direction of Glx changes might reflect multiple and complex neurobiological interactions. One possibility is that reduced thalamic Glx in poorly-controlled seizures reflects chronic medication effects. Many antiseizure medications modulate glutamatergic and GABAergic transmission to restore excitatory-inhibitory balance (Rowley et al., 2012; Costa and Vale, 2023; Sarlo and Holton, 2021; Schipper et al., 2016). The significantly lower thalamic Glx/tCr observed in patients with poorly-controlled seizures compared with patients who were seizure-free could be due to the variability in effects of monotherapy or potential interactions between antiseizure medications that influence various metabolic pathways in those undergoing polytherapy (Garcia et al., 2009; Petroff et al., 1999).

It should be noted that given the limited spatial and temporal resolution, SVS provides measurements of predominantly intracellular glutamate and glutamine levels and thus does not reflect increases in extracellular glutamate concentration related to seizures. Consequently, reduced Glx/tCr levels in poorly-controlled patients may not directly indicate absence of excitatory dysfunction but rather reflect the complex effects of medication intervention and steady-state levels in the brain. Future studies are needed to determine whether the observed Glx differences reflect seizure-related pathophysiology, medication effects, or both, to better understand network excitability and treatment response in IGEs.

Taken together, these findings reveal convergent abnormalities in cerebral perfusion and metabolism within the ACC and thalamus of patients with IGEs. The thalamus appears more susceptible to neuronal loss and metabolic abnormalities, as reflected by reduced NAA/tCr and Glx/tCr in individuals with poorly-controlled seizures. In contrast, the ACC exhibits increased perfusion that may be associated with excitatory network engagement. Together, these patterns suggest that IGEs exhibit both metabolic and perfusion alterations within the thalamocortical network. This work represents an initial step for these to potentially serve as convergent neuroimaging markers for disease characterization.

## 5 Limitations

Several limitations warrant consideration. First, the sample size was relatively small, which may have limited statistical power and generalizability of the findings, particularly regarding brain metabolite concentrations. While our atlas-based ROI approach maximizes measurement reliability, it averages across functionally distinct thalamic nuclei and ACC subregions. This averaging may attenuate nucleus- or subregion-specific perfusion differences, potentially explaining the absence of a significant thalamic CBF effect. Higher-resolution or voxel-wise perfusion analyses in larger samples are needed to resolve subregion effects. Furthermore, the use of antiseizure medications among patients with IGEs was not controlled for, which may have affected the measured metabolite concentration, and the PRESS sequence used in this study was unable to differentiate between glutamate and glutamine or detect the GABA peaks, both of which are important for understanding excitatory-inhibitory imbalance. We also implemented seizure control as a binary status and did not incorporate seizure frequency or severity; therefore, our subgroup analyses should be interpreted as reflecting broad seizure control status rather than a comprehensive index of epilepsy severity which should be considered for future studies. It should be noted that the time frame for defining “seizure-free” status in epilepsy research is variable. The current study utilized a 6-month window based on the clinical guidance of the study’s epilepsy specialists; however, prior studies have used a shorter 3-month window (Kay et al., 2013; Goodman et al., 2019), or a longer 1-year window (Capovilla et al., 2016) to define seizure-free status, and this variability should be considered when interpreting the generalizability of our seizure-free classification. Finally, while recruiting healthy controls with similarly low physical activity levels likely lead to the comparable VO_2_peak results, including a comparison group of healthy controls meeting the AHA physical activity guidelines would help determine if lifestyle factors influenced the absence of thalamic CBF differences. Future studies with larger sample sizes are needed to control for medication effects and measure both glutamate and GABA concentrations across thalamocortical network. Despite these limitations, the present multimodal investigation provides important insight into characterizing the thalamocortical network dysfunction in IGEs.

## 6 Conclusion

In summary, this multimodal investigation integrated assessments of cardiorespiratory fitness, CBF, and brain metabolites to elucidate neurobiological alterations associated with IGEs and seizure control. Cardiorespiratory fitness did not differ significantly between groups or across seizure control status. Patients with IGEs demonstrated elevated ACC CBF and reduced thalamic NAA/tCr. Particularly, patients with active seizures had lower thalamic NAA/tCr than healthy controls. We also identified a significant main effect of seizure control status on thalamic Glx/tCr ratios, with patients experiencing active seizures showing significantly lower Glx/tCr than those who were seizure-free. These findings underscore region-specific abnormalities in perfusion and neurochemistry within the thalamocortical network in IGEs. Although the influence of antiseizure medications was not explicitly examined, further research is required to clarify their impact on cerebral physiology. Overall, this study highlights the utility of combining physiological and neuroimaging methods to comprehensively characterize the pathophysiological features and seizure-control effects in IGEs.

## Data Availability

The datasets presented in this article are not readily available because data used in the current study may be made available after completion of the final data analysis for NCT04959019 and upon reasonable request from the authors. Requests to access the datasets should be directed to JBA, PhD, jallendorfer@uabmc.edu.
