# Supplementary material for: Investigating cardiorespiratory fitness, cerebral blood flow, and brain metabolism in epilepsy
Source: Front Netw Physiol. 2026 Oct 20;6:1894335. doi: 10.3389/fnetp.2026.1894335 (PMC13727442; doi:10.3389/fnetp.2026.1894335)
Supplement: Supplementary file 1 [file Table1.docx]

**Supplementary Table**

**Supplementary Table 1.** Independent t-test results and descriptive statistics (means and standard deviations) for each measure by sex.

|  | Male | Female | *t*-value | *p*-value |
| --- | --- | --- | --- | --- |
| VO_2_peak | 23.9 (7.1) | 18.9 (4.1) | –2.91 | 0.006 |
| ACC CBF | 56.0 (11.5) | 58.7 (11.5) | 0.71 | 0.482 |
| Thalamic CBF | 34.8 (9.3) | 40.9 (7.5) | 2.21 | 0.034 |
| ACC NAA/tCr | 1.2 (0.1) | 1.2 (0.1) | 0.18 | 0.862 |
| ACC Glx/tCr | 1.1 (0.1) | 1.0 (0.2) | –2.19 | 0.035 |
| Thalamic NAA/tCr | 1.4 (0.1) | 1.4 (0.2) | 1.64 | 0.110 |
| Thalamic Glx/tCr | 0.7 (0.2) | 0.7 (0.2) | 0.52 | 0.605 |
